# Supplementary material for: Management of Bilateral Congenital and Juvenile Cataracts in a Low-Income Country: Patient Identification, Treatment Outcomes, and Follow Up
Source: Children (Basel). 2024 Aug 30;11(9):1064. doi: 10.3390/children11091064 (PMC11430800; doi:10.3390/children11091064)
Supplement: Supplementary file 1 [file children-11-01064-s001.zip › children-3111281-supplementary.pdf]

Questionnaire

Question 1: What is the highest level of education of the father?

[ ] no school education

[ ] primary school

[ ] rural school

[ ] urban school

[ ] tertiary school (University)

Question 2: What is the highest level of education of the mother?

[ ] no school education

[ ] primary school

[ ] rural school

[ ] urban school

[ ] tertiary school (University)

Question 4: How would you describe your income?

[ ] Destitute

[ ] Poor

[ ] Low income

[ ] Regular income

Question 5: How was your child delivered?

[ ] Normal birth

[ ] Caesarean section

[ ] Other:

Question 6: Do you have access to water and sanitation services where you live?

[ ] Urban area with 24-hour water and sanitation services

[ ] Urban area with reduced water and sanitation access

[ ] Urban area without water and sanitation access

[ ] Rural area with 24-hour water and sanitation access

[ ] Rural area with reduced water and sanitation access

[ ] Rural area without water and sanitation access

Question 7: Is it possible for your child to attend a regular school?

[ ] Possible to attend a regular school

[ ] Not possible to attend a regular school
